# Transcendental Anatomy and Physiology

**Published:** 1845-06-01

**Authors:** 


TRANSCENDENTAL ANATOMY AND PHYSIOLOGY.—*Transcendental*, is an epithet which, of late years, has been applied by those who constitute *par-excellence* the common-sense portion of the community, to distinguish certain ultra speculatists in philosophy, sentiment and theology. To those who have been accustomed to regard the term in this its common signification, it will hardly seem possible that it can be rendered applicable to the votary of Anatomy, and Physiology. Yet we find this to be the case. M. E. R. Serres, member of the Institute of France, Professor of Natural History, &c., has published a work on what he denominates *Transcendental Anatomy applied to Physiology*. An analytical review of the work may be found in the Jan. (1845) no, of the Medico-Chirurgical Review, (London). The phrase, as this author employs it, is nearly synonymous with Embryology, and is intended to denote the study of the laws which preside over the development of the various component parts of the living organism, considered not only with reference to man, but including a comparative view of the entire animate creation.

This is a department of investigation, which has of late years become an interesting field of inquiry, not only from its intrinsic interest, but from the fact of its possessing very important relations, both to the study of healthy and morbid Physiology. For an example; (which happens to suggest itself,) in the formation of vessels in coagulable lymph effused from an inflamed surface, and its independent organization and vitality, is involved a pathological principle, exemplified physiologically in the evolution of the chick, where it is susceptible of easy demonstration. And so numerous instances might be cited. But without these practical relations, the evidences of an uniformity of the principles of constructive design, which are found to pervade the whole range of organized existences, render this province of natural history exceedingly attractive, and serve to confirm the truth, that *Nature* is but the ideal representative of an intelligent and all-wise CREATOR.

The work of M. Serres, (judging from the review in the Medico-chirurgical) must be an interesting one, and we hope will be rendered accessible in our own vernacular.

